# Depression among people living in rural and urban areas of Thailand: A cross-sectional study

**DOI:** 10.1371/journal.pone.0316077

**Published:** 2025-01-08

**Authors:** Wiriya Mahikul, Wisut Lamlertthon, Kanchana Ngaosuwan, Pawaree Nonthasaen, Napat Srisermphoak, Wares Chancharoen, Saimai Chatree, Arpaporn Arnamwong, Pisinee Narayam, Chatchamon Wandeecharassri, Pakin Wongpanawiroj

**Affiliations:** 1 Princess Srisavangavadhana College of Medicine, Chulabhorn Royal Academy, Bangkok, Thailand; 2 Chulabhorn Learning and Research Centre (CLRC), Chulabhorn Royal Academy, Bangkok, Thailand; University of Kansas School of Medicine Wichita, UNITED STATES OF AMERICA

## Abstract

**Background:**

Depression has a growing trend in the population worldwide. In this cross-sectional study, we investigated the prevalence and associated factors of depression among individuals residing in rural (Ban Luang district, Nan Province) and urban (Lak Si, Bangkok) areas of Thailand. Understanding the differences in depression between these two settings can provide insights for specific targeted interventions and mental health policies.

**Methods:**

The multistage stratified random sampling was applied to select the study participants. We recruited participants from rural and urban communities in Thailand using a structured survey questionnaire through either face-to-face interviews or in paper or electronic form. We collected data on depression using the Patient Health Questionnaire-9 (PHQ-9) tool and sociodemographic characteristics and conducted descriptive statistics and logistic regression analysis.

**Results:**

Of 867 survey participants, 420 were from rural areas (Nan) and 447 were from urban areas (Bangkok). Participants’ mean age was 55.9±9.5 years in rural areas and 56.0±12.0 years in urban areas. Most participants in urban areas were women, married, and had lower education levels (71.1%, 50.3%, 58.8%, respectively). The overall prevalence of depression across both settings was 18.6%. We found a higher prevalence of depression in the urban (31.8%) than the rural (4.5%) setting. In multiple logistic regression analysis, urban residence was significantly associated with higher rates of depression compared with rural residence (adjusted odds ratio [AOR] 9.43, 95% confidence interval [CI] 5.08–17.52). Nuclear family and using social media were associated with lower levels of depression in urban areas (AOR 0.50 and 0.43, 95% CI 0.27–0.93 and 0.22–0.84, respectively). Higher education level was significantly associated with higher levels of depression in rural areas (AOR 3.84, 95% CI 1.19–12.42).

**Conclusion:**

This study emphasized the difference in depression and related factors between rural and urban areas of Thailand, highlighting a greater prevalence in urban areas. To help prevent depression, it is important to address specific challenges in each setting, such as those faced by highly educated individuals living in rural areas with high depression rates, exploring social media use patterns in urban populations, and understanding dynamics of the nuclear family. Our findings can inform the development of public health policies aimed at effectively mitigating the burden of depression and improving overall mental well-being in specific settings.

## Introduction

Depression is a serious global public health concern affecting millions of individuals worldwide, with detrimental effects on quality of life, productivity, and overall well-being [1]. In Thailand, as in many other countries, the burden of depression is a growing issue that warrants attention and understanding [2]. Social factors are significant risk contributors to depression [3], such as marital status [4], and educational attainment [5]. Additionally, behaviors like smoking and alcohol consumption are well-established risk factors for depression [6, 7]. Furthermore, studies have shown that the area of residence may also be associated with an increased risk of depression [8, 9]. Exploring the prevalence and factors associated with depression among individuals residing in different geographical areas, such as in rural and urban settings, can provide valuable insights into the varying contexts and potential contributing factors of depression [10].

Differences in rural and urban settings are associated with differences in the depression rates in several countries [11–20]. Rural areas often possess distinct characteristics that could eventually lead to depressive mental illnesses [11–13, 18]. The rise in depression cases with older age and certain disadvantages in rural settings is largely influenced by sociodemographic factors and physical disabilities [18]. Residence in rural areas is associated with depression, which, in turn, is associated with thoughts of suicide [20]. A systematic review in low-to-middle income countries found consistent links between variables such as poverty, food insecurity, housing or education, and depression in both rural and urban areas [21]. These factors may contribute to the different landscapes of depression and its associated risk factors. For instance, urban areas typically face challenges such as violence, homelessness, mass media exposure, a fast-moving lifestyle, and excessive workloads, which have a relationship with increased rates of mental disorders [22]. Understanding the variations in the depression prevalence between these two settings is crucial for tailoring effective interventions and public health strategies. By applying a cross-sectional design, the present study aim is to provide a snapshot analysis of the depression rates in both rural and urban areas of Thailand, providing a broader understanding of the mental health challenges faced by individuals throughout Thailand. Furthermore, identifying the factors associated with depression in these different contexts can help guide targeted interventions and policies aimed at reducing the burden of depression and improving overall mental well-being.

This research constitutes a component of “Princess Chulabhorn’s Health Volunteer Project: A Medical Integration and Innovation for Sustainable Communities.” The project was initiated by Her Royal Highness Princess Chulabhorn Krom Phra Srisavangavadhana with the primary objective of enhancing the overall well-being of the Thai population. To achieve this goal, we collected health data in this study, with a particular focus on depression. Our findings will not only contribute to the existing body of knowledge regarding depression in Thailand but will also have implications for mental health policies and interventions in both rural and urban areas. It is hoped that the insights gained from the present research will support the development of tailored and context-specific strategies to address depression and enhance the overall mental health of the Thai population in specific settings.

## Materials and methods

### Study design

**Fig 1:**
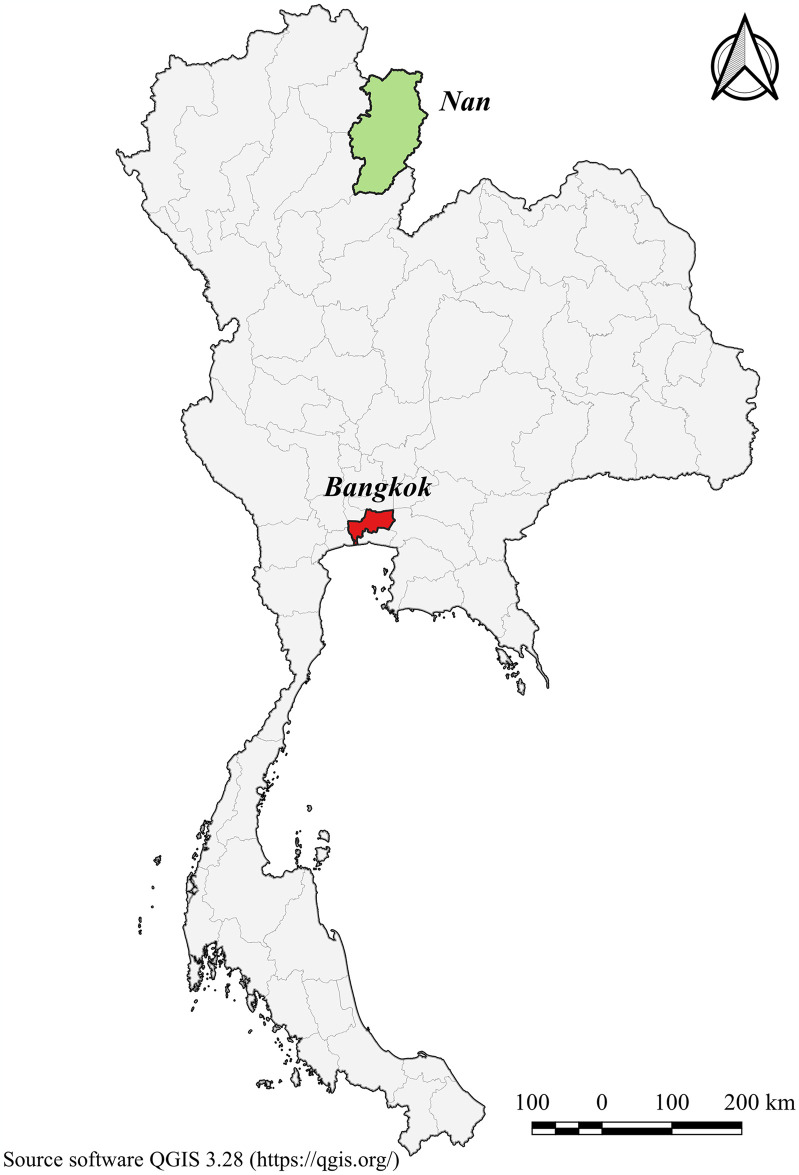
Map of Nan (northern region) and Bangkok (central region) in Thailand.

This study followed a cross-sectional study design, which allows for the collection of data at a specific point in time. The study design enabled us to investigate the prevalence of depression as well as its associated factors among individuals living in rural (Ban Luang, Nan) and urban (Lak Si, Bangkok) areas of Thailand, depicted in Fig 1. The study used a quantitative research design to collect data from a representative sample of participants using standardized assessment tools. The study includes a comprehensive analysis of sociodemographic factors and lifestyle characteristics with the aim to better understand the contextual factors contributing to depression in different geographic settings.

### Study site

In this cross-sectional study, we aimed to investigate the prevalence of depression in Thailand among people living in a rural area (Ban Luang district in Nan Province, where transportation is far from the city center) as well as among residents of an urban area (Lak Si in Bangkok, which is close to Don Muang Airport and has large buildings and shopping malls). Nan is among the 77 provinces of Thailand and lies in the northern part of the country. Its landscape primarily comprises mountains and forests with an area of 12,163.04 square kilometers. The population of Nan is a diverse mix of 478,227 individuals, including ethnic minorities and Thai residents [23]. The primary economic focus of Nan is the agricultural sector, contributing 29% to its gross provincial product (GPP) [24]. Additionally, 12% of GPP is attributed to the detailing sector, and educational activities account for 11%. Bangkok is the capital city of Thailand and is the most densely populated area of the country, boasting approximately 5.49 million registered residents in 2023 within an area of only 1,568.73 square kilometers [25, 26]. Bangkok primarily comprises the city and urbanizations [26]. Most of Bangkok’s GPP involves the detailing sector, with approximately 22% of total GPP. Financial and insurance activities follow at 14%, and agricultural activities account for the smallest share at 0.04% [24]. By comparing these two distinct settings, we aimed to discern potential differences in the prevalence rates of depression and examine the influence of various factors unique to each area.

### Sampling

To recruit participants from both areas, we used a multistage stratified random sampling technique. In the first stage, random selection was conducted to choose districts and provinces that are representative of rural and urban areas (rural: Ban Luang district, Nan, urban: Lak Si district, Bangkok). In the second stage, random sampling was used to select subdistricts within each chosen district. Finally, in the third stage, households within the selected subdistricts were randomly sampled. The inclusion criteria for this study encompassed individuals aged 20 years or older who were able to communicate effectively in Thai language. Individuals who met the inclusion criteria within each household were then identified and invited to participate in the study. Participants with incomplete data on the PHQ-9 and covariates were excluded. Data sampling for this cross-sectional study was carried out across two distinct periods: 2–3 July 2022 in Lak Si, Bangkok and 6–10 March 2023 in Ban Luang, Nan. The target population comprised individuals residing in rural (Nan) and urban (Bangkok) areas of Thailand.

### Sample size

The sample size for this study was calculated based on the estimated prevalence of depression of 12% [27], anticipated effect size (p), and desired level of precision (d). To estimate the prevalence of depression, the formula for an infinite population proportion (n = Z^2^p(1−p)/d^2^) was used to calculate the sample size This calculation ensured an adequate sample size to detect the prevalence of depression in both rural and urban areas with a precision of 2.5% (d) and a 95% confidence level. Consequently, the minimum calculated sample size was 650. Accounting for a 25% non-response rate, the maximum calculated sample size was approximately 813. Therefore, our sample comprised 867 participants, with 420 individuals from rural areas (Nan) and 447 individuals from urban areas (Bangkok) (S1 Data).

### Data collection

After obtaining informed consent from each participant prior to data collection, trained personnel were assigned to facilitate the administration of a structured survey questionnaire through either face-to-face interviews or in paper or electronic form (google form), depending on participants’ preference. Literate participants could choose to complete either the paper version or, if comfortable with technology, the electronic form (google form) on a mobile phone. For elderly and illiterate participants, who were generally unable to use these methods, the scale was administered through face-to-face interviews with an interviewer. We engaged village health volunteers (VHVs) to serve as representatives for collecting questionnaire data from residents. A total of 25 VHVs collaborated with our team and through training by the research staff on the data collection process, review procedures, and return protocols before approaching participants. After ensuring that all VHVs were proficient in the questionnaire collection methods, they were assigned to their designated areas to randomly select participants based on the established criteria. Once the selection process was confirmed, the VHVs returned the completed questionnaires to the researchers. We then conducted a thorough review of the questionnaires for accuracy and completeness. If any discrepancies or errors were found, we engaged with the relevant VHVs to correct or verify the data.

This comprehensive survey encompassed four key sections, the first of which included sociodemographic information, in which data concerning participants’ age, sex, marital status, education level, average household monthly income (THB), occupation, type of house, type of family, place of residence, and underlying diseases were identified through self-reports of having received a diagnosis from a physician. Age in years was recategorized and stratified into two categories, age <60, and ≥60 years. Sex was coded as male or female. Marital status was coded as married, unmarried, or divorced. Education level was categorized as uneducated (no formal education), lower education level (below a bachelor’s degree, including primary, secondary, or vocational education), bachelor’s degree (completed undergraduate studies), and higher education level (education above a bachelor’s degree, such as master’s or doctoral degrees), based on the highest level attained. Average household monthly income was coded as <10,000 THB and ≥10,000 THB. Occupation was coded as unemployed, farmer, non-government employee, and government employee. The type of house was coded as twin house, village house, or condominium. The family type was coded as extended family or nuclear family. Place of residence was coded as either urban or rural. Underlying diseases, including chronic conditions like hypertension and diabetes, were coded as "yes" for presence and "no" for absence.

The section on lifestyle factors, whether daily, occasional, or one-time, included questions about participants’ social media use, smoking, and alcohol consumption. Participants were asked to provide yes/no answers to a series of questions regarding their engagement in social media use (yes for those who have ever used it, no for those who have never used it), smoking (yes for those who have smoked, no for those who have never smoked), and drinking alcohol (yes for those who have consumed it, no for those who have never consumed it) within the past six months. All of the aforementioned variables were considered independent variables in this study.

The method of depression assessment used in this study was a standardized self-assessment tool, the Patient Health Questionnaire-9 (PHQ-9) [28]. The validated Thai version of the PHQ-9, developed by Lotrakul, M., Sumrithe, S., and Saipanish, R. (2008), was employed in this study [29]. This was the dependent variable in this analysis. The PHQ-9 is a nine-item questionnaire screening tool for identifying and evaluating the severity of depression, with each item corresponding to one of nine criteria for diagnosing major depressive disorders based on the Diagnostic and Statistical Manual of Mental Disorders-IV (DSM-IV). This assessment tool demonstrated robust test—retest reliability for psychiatric disorders, with a coefficient of 0.85, indicating its consistent performance over time in our study. Additionally, the PHQ-9 has high sensitivity and specificity, with rates of 84% and 97%, respectively [30].

Participants were asked about mental health symptoms that they may have experienced over the previous 2 weeks and were asked to give a frequency score of 0 to 3 for each of the nine items, where 0 is “not at all” and 3 is “nearly every day.” Scores for each item are summed to obtain a total score, ranging from 0 to 27. The total score is then used to gauge the severity of depressive symptoms, with scores of 0 to 4 indicating no depression. Scores of 5 or higher are considered indicative of possible depressive symptoms, which are categorized as follows: 5–9 indicates mild depression, 10–14 indicates moderate depression, 15–19 indicates moderately severe depression, and 20–27 indicates the presence of severe depression [28]. The PHQ-9 is a validated tool for mental health screening, as confirmed in several studies [31, 32] and has been successfully applied to prior research to assess depression in rural Australian populations [33, 34].

### Data analysis

Descriptive statistics, such as frequency, percentage, mean, and standard deviation (SD), were used to perform descriptive analyses and summarize the sociodemographic characteristics, prevalence, and severity of depression, as well as lifestyle factors. A comparative analysis was performed using the chi-squared (χ^2^) test to compare depression and factors associated with residence in urban and rural areas. The outcome we focus on in our binary logistic regression analysis is depression, categorized as "yes" for a score of 5 or higher and "no" for a score below 5 using PHQ-9. To further test these associations, univariate logistic regression analysis was performed to identify independent factors associated with depression in both rural and urban settings, as well as separately for each. The odds ratio (OR) and corresponding 95% confidence interval (CI) were obtained. Variables with P-values <0.05 in the univariate analysis were retained and used in a multiple logistic regression analysis with adjusted odd ratios (AOR). This statistical measure is modified to account for the influence of additional predictor variables, adjusting for potential confounders such as sociodemographic factors and lifestyle characteristics. Variables with a P-value <0.05 were considered statistically significant. IBM SPSS version 28 (IBM Corp., Armonk, NY, USA) was used to perform all statistical analyses.

### Ethical statement

This study was approved by the Human Research Ethics Committee, Chulabhorn Royal Academy, Thailand (EC-195/2564), and it conformed to the ethics guidelines of the Declaration of Helsinki. Further approval was obtained from the general community before the commencement of this study. Written informed consent was obtained from all participants and if the subjects are illiterate, informed consent was obtained from legal representative. This study is reported in compliance with the STROBE statement.

## Results

### Sample characteristics

This study included 867 participants, with 420 individuals from rural areas (Nan) and 447 individuals from urban areas (Bangkok). The mean age of participants was 55.9 years (SD = 9.5) in rural areas and 56.0 years (SD = 12.0) in urban areas. There were statistically significant differences between demographic and lifestyle factors and residing in a rural (Nan) or urban (Bangkok) area of Thailand, with P-values <0.05, except for age group, where the population aged 60 years and older comprised 34.5% in rural areas and 40.5% in urban areas (P = 0.07), as shown in Table 1. Most participants were women, 61.7% and 71.1% in rural and urban areas, respectively. In rural areas, 84.3% of people had education levels below a bachelor’s degree, and 8.6% had no education; in urban areas, these percentages were 58.8% and 1.6%, respectively. Income levels were generally lower in rural areas, with 90% of participants reporting a monthly income below the national poverty line (<10,000 THB), compared with 30.2% in urban areas. Significant differences were found in employment status between the two areas, with most participants engaged in non-government employment or farming in both urban (70.0%) and rural (90.0%) areas. The housing contrast was striking, with 99.8% of rural residents living in village houses and 90.2% of the urban population residing in a condominium. Family type showed no significant variation, with nuclear families prevalent in both urban (89.3%) and rural (83.1%) areas. In urban areas, half of participants (52.3%) had underlying diseases; rural areas showed a similar percentage at 45.0%. Social media use varied, with 91.5% of urban residents and 70.5% of rural residents engaging in social media. A small proportion of urban residents (15.7%) smoked; in rural areas, the proportion was greater (22.9%). Notably, a sharp contrast in alcohol consumption was evident, with 65.7% of the rural population reporting alcohol intake compared with 34% in the urban population.

**Table 1:** Demographic characteristics of participants (N = 867), with 420 rural residents (Nan) and 447 urban residents (Bangkok).

Variables	Urban (n = 447, 51.6%)	Rural (n = 420, 48.4%)	Total (N = 867)	P-value
Sex				
Female	318 (71.1)	259 (61.7)	577 (66.6)	**0.003**
Male	129 (28.9)	161 (38.3)	290 (33.4)	
Age (years)				
<60	266 (59.5)	275 (65.5)	541 (62.4)	0.070
≥60	181 (40.5)	145 (34.5)	326 (37.6)	
Status				
Unmarried	146 (32.7)	19 (4.5)	165 (19.0)	**<0.001**
Married	225 (50.3)	357 (85.0)	582 (67.2)	
Divorced	76 (17.0)	44 (10.5)	120 (13.8)	
Education level				
Uneducated	7 (1.6)	36 (8.6)	43 (5.0)	**<0.001**
Lower education level (below bachelor’s degree)	263 (58.8)	354 (84.3)	617 (71.1)	
Bachelor’s degree	155 (34.7)	22 (5.2)	177 (20.4)	
Higher education level (above bachelor’s degree)	22 (4.9)	8 (1.9)	30 (3.5)	
Average household monthly income (THB)				
<10,000	135 (30.2)	378 (90.0)	513 (59.2)	**<0.001**
≥10,000	312 (69.8)	42 (10.0)	354 (40.8)	
Occupation				
Unemployed	69 (15.5)	28 (6.7)	97 (11.2)	**<0.001**
Non-government employee/ farmer	313 (70.0)	378 (90.0)	691 (79.7)	
Government employee	65 (14.5)	14 (3.3)	79 (9.1)	
Type of house				
Twin house	11 (2.5)	0 (0.0)	11 (1.3)	**<0.001**
Village house	33 (7.4)	419 (99.8)	452 (52.1)	
Condominium	403 (90.1)	1 (0.2)	404 (46.6)	
Type of family				
Extended family	48 (10.7)	71 (16.9)	119 (13.7)	**0.008**
Nuclear family	399 (89.3)	349 (83.1)	748 (86.3)	
Underlying diseases				
No	213 (47.7)	231 (55.0)	444 (51.2)	**0.031**
Yes	234 (52.3)	189 (45.0)	423 (48.8)	
Social media use				
No	38 (8.5)	124 (29.5)	162 (18.7)	**<0.001**
Yes	409 (91.5)	296 (70.5)	705 (81.3)	
Smoking				
No	377 (84.3)	324 (77.1)	701 (80.9)	**0.007**
Yes	70 (15.7)	96 (22.9)	166 (19.1)	
Alcohol consumption				
No	295 (66.0)	144 (34.3)	439 (50.6)	**<0.001**
Yes	152 (34.0)	276 (65.7)	428 (49.4)	
Depression				
No (<5)	305 (68.2)	401 (95.5)	706 (81.4)	**<0.001**
Yes (≥5)	142 (31.8)	19 (4.5)	161 (18.6)	
Depression severity				
No symptoms (<5)	305 (68.2)	401 (95.5)	706 (81.4)	**<0.001**
Mild (5–9)	101 (22.6)	18 (4.3)	119 (13.7)	
Moderate (10–14)	29 (6.5)	1 (0.2)	30 (3.5)	
Severe (15–19)	9 (2.0)	0 (0.0)	9 (1.0)	
Moderately severe (≥20)	3 (0.7)	0 (0.0)	3 (0.4)	

Values in the table are n (%). P-values were calculated using chi-square test.

### Prevalence of depression

The overall prevalence of depression across both settings was 18.6%. The prevalence of depression among individuals in rural areas was 4.5%; in urban areas, this was 31.8% (P-value <0.001), as shown in Table 1. No individuals in rural areas experienced severe or moderately severe depression, compared with urban areas where the rates were 2.0% and 0.7%, respectively. Table 2 presents the number of participants answering each PHQ-9 question. The data illustrate that the urban population scored significantly higher for each item compared with the rural population (P<0.001), with some remarkable observations. For instance, 27 urban residents (6%) reported trouble sleeping nearly every day compared with only three rural residents (0.7%). Additionally, 20 urban respondent (4.5%) reported having a poor appetite or overeating nearly every day versus only one rural respondent (0.2%).

**Table 2:** Responses to the questionnaire on depression in Bangkok and Nan, Thailand.

Statement	Nearly every day		More than half of days		Several days		Not at all		P-value
	Urban n (%)	Rural n (%)	Urban n (%)	Rural n (%)	Urban n (%)	Rural n (%)	Urban n (%)	Rural n (%)	
1. Little interest or pleasure in doing things	3 (0.7)	0 (0.0)	17 (3.8)	0 (0.0)	113 (25.3)	35 (8.3)	314 (70.2)	385 (91.7)	**<0.001**
2. Feeling down, depressed, or hopeless	3 (0.7)	0 (0.0)	15 (3.4)	0 (0.0)	87 (19.5)	23 (5.5)	342 (76.5)	397 (94.5)	**<0.001**
3. Trouble falling or staying asleep, or sleeping too much	27 (6.0)	3 (0.7)	60 (13.4)	10 (2.4)	177 (39.6)	121(28.8)	183 (40.9)	286 (68.1)	**<0.001**
4. Feeling tired or having little energy	11 (2.5)	0 (0.0)	35 (7.8)	0 (0.0)	138 (30.9)	51 (12.1)	263 (58.8)	369 (87.9)	**<0.001**
5. Poor appetite or overeating	20 (4.5)	1 (0.2)	39 (8.7)	1 (0.2)	156 (34.9)	44 (10.5)	232 (51.9)	374 (89.0)	**<0.001**
6. Feeling bad about yourself or feeling a failure or that have let yourself or your family down	7 (1.6)	0 (0.0)	24 (5.4)	1 (0.2)	66 (14.8)	14 (3.3)	350 (78.3)	405 (96.4)	**<0.001**
7. Trouble concentrating on things, such as reading the newspaper or watching television	10 (2.2)	0 (0.0)	29 (6.5)	1 (0.2)	112 (25.1)	34 (8.1)	296 (66.2)	385 (91.7)	**<0.001**
8. Moving or speaking so slowly that other people have noticed, or being so fidgety or restless that you have been moving around a lot more than usual	4 (0.9)	1 (0.2)	20 (4.5)	1 (0.2)	72 (16.1)	13 (3.1)	351 (78.5)	405 (96.4)	**<0.001**
9. Thoughts that you would be better off dead or of hurting yourself in some way	2 (0.4)	1 (0.2)	8 (1.8)	0 (0.0)	24 (5.4)	3 (0.7)	413 (92.4)	416 (99.0)	**<0.001**

Values in the table are n (%). P values were calculated using chi-square test.

### Factors associated with depression

We found an association between the residential area and depression. Multiple logistic regression analysis showed that the urban population had an approximately nine times higher risk of having depression compared with the rural population (AOR = 9.43, 95% CI 5.08–17.52, P<0.001), as shown in Table 3. The table shows the results of multiple logistic regression analysis for the association between depression and independent variables in both settings. In rural areas, people with education levels above a bachelor’s degree had an approximately four times higher risk of depression compared with people with education levels below a bachelor’s degree (AOR = 3.84, 95% CI 1.19–12.42, P = 0.024), as shown in Table 4. In the urban population, the type of family structure appeared to mitigate the risk of depression. Individuals residing in nuclear families had an approximately 47% lower risk of developing depression compared with those with an extended family structure (AOR = 0.53, 95% CI 0.28–0.98, P = 0.044). The use of social media was also found to be a protective factor against depression. Urban respondents who reported using social media had an approximately 55% lower risk of depression compared with those not using social media (AOR = 0.45, 95% CI 0.22–0.88, P = 0.020).

**Table 3:** Multiple logistic regression models for the relationship between depression (PHQ-9 ≥ 5) and independent variables, Thailand.

Variable	Category	Depression							
		No (< 5) n (%)	Yes (≥ 5) n (%)	OR	95% CI	P-value	AOR	95% CI	P-value
Residential area	Rural	401 (56.8)	19 (11.8)	Ref.					
	Urban	305 (43.2)	142 (88.2)	9.82	5.95–16.22	**<0.001**	9.43	5.08–17.52	**<0.001**
Sex	Female	465 (65.9)	112 (69.6)	Ref.					
	Male	241 (34.1)	49 (30.4)	0.84	0.58–1.22	0.369			
Age (years)	<60	436 (61.8)	105 (65.2)	Ref.					
	≥60	270 (38.2)	56 (34.8)	0.86	0.60–1.23	0.414			
Status	Unmarried	119 (16.9)	46 (28.6)	Ref.					
	Married	499 (70.7)	83 (51.6)	0.43	0.28–0.65	**<0.001**	0.99	0.62–1.56	0.968
	Divorced	88 (12.5)	32 (19.9)	0.94	0.55–1.59	0.821	1.53	0.84–2.74	0.159
Education level	Uneducated	40 (5.7)	3 (1.9)	Ref.					
	Lower education level (below bachelor’s degree)	520 (73.7)	97 (60.2)	2.49	0.75–8.20	0.134	1.62	0.44–5.95	0.465
	Bachelor’s degree	120 (17.0)	57 (35.4)	6.33	1.87–21.34	**0.003**	2.34	0.59–9.17	0.222
	Higher education level (above bachelor’s degree)	26 (3.7)	4 (2.5)	2.05	0.42–9.92	0.372	0.89	0.16–5.04	0.904
Average household monthly income (THB)	<10,000	450 (63.7)	63 (39.1)	Ref.					
	≥10,000	256 (36.3)	98 (60.9)	2.73	1.92–3.89	**<0.001**	0.89	0.55–1.45	0.650
Occupation	Unemployed	71 (10.1)	26 (16.1)	Ref.					
	Farmer or non-government employee	577 (81.7)	114 (70.8)	0.54	0.33–0.88	**0.014**	0.81	0.46–1.45	0.479
	Government employee	58 (8.2)	21 (13.0)	0.98	0.50–1.93	0.974	0.79	0.36–1.79	0.585
Type of house	Twin house	10 (1.4)	1 (0.6)	Ref.					
	Village house	423 (59.9)	29 (18.0)	0.68	0.08–5.54	0.723			
	Condominium	273 (38.7)	131 (81.4)	4.79	0.60–37.88	0.137			
Type of family	Extended family	94 (13.3)	25 (15.5)	Ref.					
	Nuclear family	612 (86.7)	136 (84.5)	0.83	0.51–1.34	0.462			
Social media use	No	139 (19.7)	23 (14.3)	Ref.					
	Yes	567 (80.3)	138 (85.7)	1.47	0.91–2.37	0.114			
Underlying diseases	No	360 (51.0)	84 (52.2)	Ref.					
	Yes	346 (49.0)	77 (47.8)	0.95	0.68–1.34	0.787			
Smoking	No	567 (80.3)	134 (83.2)	Ref.					
	Yes	139 (19.7)	27 (16.8)	0.82	0.52–1.29	0.396			
Alcohol consumption	No	342 (48.4)	97 (60.2)	Ref.					
	Yes	364 (51.6)	64 (39.8)	0.62	0.43–0.87	**0.007**	1.19	0.79–1.77	0.405

CI, confidence interval; OR, odds ratio; AOR, adjusted odds ratio.

**Table 4:** Multiple logistic regression models for the relationship between depression (PHQ-9 ≥ 5) and independent variables in urban and rural Thailand.

Variable	Category	Depression in urban residents						Depression in rural residents					
		No (< 5) n (%)	Yes (≥ 5) n (%)	OR (95% CI)	P-value	AOR (95% CI)	P-value	No (< 5) n (%)	Yes (≥ 5) n (%)	OR (95% CI)	P-value	AOR (95% CI)	P-value
Sex	Female	218 (71.5)	100 (70.4)	Ref.				247 (61.6)	12 (63.2)	Ref.			
	Male	87 (28.5)	42 (29.6)	1.05 (0.67–1.63)	0.819			154 (38.4)	7 (36.8)	0.93 (0.36–2.42)	0.891		
Age (years)	<60	174 (57.0)	92 (64.8)	Ref.				262 (65.3)	13 (68.4)	Ref.			
	≥60	131 (43.0)	50 (35.2)	0.72 (0.47–1.09)	0.121			139 (34.7)	6 (31.6)	0.87 (0.32–1.33)	0.782		
Status	Unmarried	102 (33.4)	44 (31.0)	Ref.				17 (4.2)	2 (10.5)	Ref.			
	Married	156 (51.1)	69 (48.6)	1.02 (0.65–1.61)	0.914			343 (85.5)	14 (73.7)	0.34 (0.07–1.65)	0.183		
	Divorced	47 (15.4)	29 (20.4)	1.43 (0.79–2.56)	0.228			41 (10.2)	3 (15.8)	0.62 (0.09–4.06)	0.620		
Education level	Lower (uneducated and below bachelor’s degree)	185 (60.7)	85 (59.9)	Ref.				375 (93.5)	15 (78.9)	Ref.		Ref.	
	Higher (bachelor’s degree and above bachelor’s degree)	120 (39.3)	57 (40.1)	1.03 (0.68–1.55)	0.873			26 (6.5)	4 (21.1)	3.84 (1.19–12.42)	**0.024**	3.84 (1.19–12.42)	**0.024**
Average household monthly income (THB)	<10,000	89 (29.2)	46 (32.4)	Ref.				361 (90.0)	17 (89.5)	Ref.			
	≥10,000	216 (70.8)	96 (67.6)	0.86 (0.56–1.32)	0.491			40 (10.0)	2 (10.5)	1.06 (0.23–4.76)	0.938		
Occupation	Unemployed	43 (14.1)	26 (18.3)	Ref.				28 (7.0)	0 (0.0)	-			-
	Farmer or non-government employee	218 (71.5)	95 (66.9)	0.72 (0.41–1.24)	0.237			359 (89.5)	19 (100.0)	-			-
	Government employee	44 (14.4)	21 (14.8)	0.78 (0.38–1.60)	0.515			14 (3.5)	0 (0.0)	-			-
Type of house	Twin house	10 (3.3)	1 (0.7)	Ref.				0 (0.0)	0 (0.0)	-			-
	Village house	23 (7.5)	10 (7.0)	4.34 (0.48–38.67)	0.188			400 (99.8)	19 (100.0)	-			-
	Condominium	272 (89.2)	131 (92.3)	4.81 (0.61–38.02)	0.136			1 (0.2)	0 (0.0)	-			-
Type of family	Extended family	26 (8.5)	22 (15.5)	Ref.				68 (17.0)	3 (15.8)	Ref.			
	Nuclear family	279 (91.5)	120 (84.5)	0.50 (0.27–0.93)	**0.029**	0.53 (0.28–0.98)	**0.044**	333 (83.0)	16 (84.2)	1.08 (0.30–3.84)	0.894		
Social media use	No	19 (6.2)	19 (13.4)	Ref.				120 (29.9)	4 (21.1)	Ref.			
	Yes	286 (93.8)	123 (86.6)	0.43 (0.22–0.84)	**0.014**	0.45 (0.22–0.88)	**0.020**	281 (70.1)	15 (78.9)	1.60 (0.52–4.92)	0.411		
Underlying diseases	No	140 (45.9)	73 (51.4)	Ref.				220 (54.9)	11 (57.9)	Ref.			
	Yes	165 (54.1)	69 (48.6)	0.80 (0.53–1.19)	0.278			181 (45.1)	8 (42.1)	0.88 (0.34–2.24)	0.795		
Smoking	No	259 (84.9)	118 (83.1)	Ref.				308 (76.8)	16 (84.2)	Ref.			
	Yes	46 (15.1)	24 (16.9)	1.14 (0.66–1.96)	0.622			93 (23.2)	3 (15.8)	0.62 (0.17–2.17)	0.457		
Alcohol consumption	No	207 (67.9)	88 (62.0)	Ref.				135 (33.7)	9 (47.4)	Ref.			
	Yes	98 (32.1)	54 (38.0)	1.29 (0.85–1.96)	0.221			266 (66.3)	10 (52.6)	0.56 (0.22–1.42)	0.224		

CI, confidence interval; OR, odds ratio; AOR, adjusted odds ratio.

## Discussion

In this study, we sought to examine the difference in prevalence and factors associated with depression in two distinctive regions of Thailand: Nan, which represents a rural setting, and Bangkok, representative of an urban setting. Using the PHQ-9, we assessed the prevalence and severity of depression and associated factors such as demographic characteristic, lifestyle variables, and other relevant information. The overall prevalence of depression across both settings was 18.6%, which is consistent with previous studies [27, 35]. We found a higher prevalence of depression in the urban (31.8%) than the rural (4.5%) setting, which is consistent with previous studies in other countries [36, 37], although some studies contradict this finding [13]. We found an approximately nine times higher prevalence of depression in the urban area versus the rural area (OR 9.43, P<0.001), which is consistent with previous studies [12, 38]. This may be owing to the influence of sociocultural aspects of urban living, such as disorganization and quality of the physical environment, on mental health, as described in some research [39, 40]. In contrast, other studies have shown that living in a rural area increased the likelihood of experiencing depression as compared with an urban setting [11–13]. Additionally, studies indicated that the unadjusted prevalence of depression was significantly higher among rural populations than among urban populations [18, 41]. This is possibly owing to differing population characteristics, such as low socioeconomic status and disparities in access to hospitals [42]. This discrepancy may be explained by differences in the sociocultural, economic, and healthcare contexts between Thailand and other countries. In many countries where rural populations showed higher depression levels, rural areas often face significant challenges, such as limited access to healthcare, including mental health services, and economic hardships exacerbated by the pandemic [11–13]. These factors can contribute to higher levels of depression. However, in Thailand, rural areas may have benefited from stronger community ties, cultural norms emphasizing collective support, and a slower pace of life, which could have mitigated the psychological impact of the pandemic. Moreover, rural areas in Thailand might have been less affected by strict lockdown measures and economic disruptions compared to urban areas, where industries and services were more severely impacted. Following logistic regression analysis, our findings indicated that within urban areas, the presence of a nuclear family structure served as a protective factor against depression. This observation aligns with prior research highlighting the important influence of the family structure on mental health outcomes of its members [43]. According to evidence from China and India, plausible explanations for the lower rates of depression in nuclear families include a reduced financial burden on the younger generation, thereby mitigating stress and minimizing interpersonal tensions, which could consequently contribute to less work—family conflict, and enhancing mental well-being [44, 45]. Conversely, some researchers posit an increased risk of depression within nuclear families, particularly attributable to an absence of emotional support or caregiving mechanisms [46]. Further research is needed on which specific characteristics of the nuclear family structure can contribute to the amelioration of mental health within the Thai population.

This study additionally revealed that the utilization of social media may serve as a protective factor against depression in urban areas. Numerous investigations have delved into the intricate relationship between depression and social media engagement [47–51]. Cross-sectional studies indicate an elevated incidence of depression associated with social media use [49, 50]. This phenomenon may be elucidated through an abductive biopsychological model, implicating obligatory engagement irrespective of adverse consequences [48]. Contrarily, a longitudinal study suggested an inverse relationship, asserting that social media use does not precipitate depressive symptoms; instead, depression predisposes individuals to heightened social media engagement [47]. Concurrently, compelling evidence suggests that social media utilization could function as an indicator of impending depression, facilitating early detection [51]. In this study, we discovered a protective influence of social media use against depression within the urban population of Bangkok. This finding is potentially elucidated by the capacity of social media to enhance social integration and foster social identity development, ultimately contributing to the overall well-being of individuals in the urban context [52]. Furthermore, the recreational facet of engaging with social media might play a role in reducing the incidence of depression in urban areas. Consequently, on the basis of the outcomes of this study, we advocate further exploration to identify specific functions of social media use that may be instrumental in preventing depression among the urban population of Thailand.

In the rural population, we found that more highly educated participants (above a bachelor’s degree) were more likely to develop depression. According to past research, education level is associated with depression and other mental disorders, such as anxiety [53]. Furthermore, higher education levels are recognized as a protective factor against depression in many studies, particularly in people with disadvantaged backgrounds [54, 55]. Research suggests that education may prevent depression by providing increased economic resources, thereby enhancing social status and overall health [56]. However, our study in rural Thailand contradicts this notion, revealing that educated residents of rural areas like Nan experienced a higher rate of depression. Despite a higher education, individuals in rural areas struggle to secure high-income jobs, as reflected in the GPP and derivatives in rural regions, as compared with urban centers like Bangkok [57]. Even with higher education levels, individuals facing economic disadvantages may not perceive themselves as being in control, which leads to unwarranted self-blame for their circumstances [58]. If conditions among lower-income groups do not facilitate a feeling of having one’s life circumstances under control, this can result in disappointment and frustration owing to external obstacles and constraints [59]. Conversely, scholarly investigations suggest that an elevated sense of control can serve as a mediator of depressive symptoms [55, 60]. Our findings imply the need for additional research in the population with higher education levels residing in rural areas. Identifying their challenges and implementing targeted policies could assist this specific group in preventing the onset of depression.

### Strengths and limitations

The present study had notable strengths. First, the study locations in Thailand enhance the relevance and contextual applicability of the findings to the specific sociocultural dynamics of each region. Additionally, the inclusion of a substantial number of participants contributes to the statistical reliability of the study and observed patterns. Furthermore, the deliberate inclusion of rural areas like Nan, which may face logistical challenges in terms of access, adds value to the research by providing insights into populations that are often underrepresented in scientific research. These strengths enhance the study’s credibility and broaden our understanding of the investigated phenomena.

This study also had several limitations. First, the findings are based on cross-sectional data, which restrict the ability to establish causal relationships between variables. Second, we concentrated on specific rural (Nan) and urban (Bangkok) areas, thereby constraining the generalizability of the results to broader geographic regions. Thirdly, the survey was conducted across different time periods in two distinct settings, with the time periods chosen due to limited access to the rural community. During the harvest season, from July until the end of the year, farmers are very busy with agricultural activities. Therefore, we decided to conduct the survey in March, when the rural population was more available. The data was collected during two periods, both during and after the COVID-19 pandemic, which may influence the mental health outcomes. Fourth, most of the participants are women, so the results may be less representative of all sexes. Fifth, the imbalance in the prevalence of depression in each setting may result in greater variation in the confidence interval. Lastly, the reliance on self-report measures introduces the potential for response bias because participants may provide information based on subjective interpretations or social desirability. Future research should consider longitudinal designs, diverse geographical representations, and a mix of assessment methods to enhance applicability of the findings.

## Conclusions

This study highlighted the importance of distinctions between rural and urban populations in identifying factors contributing to the prevalence of depression. We propose further inquiry to address challenges faced by the population in rural areas with higher education levels, which potentially contribute to an elevated prevalence of depression. Additionally, in urban areas, elucidating specific patterns or features of social media use may aid in implementing strategies to prevent depression. Identifying distinctive patterns of family relationships and interactions within nuclear families in urban areas could strengthen the family unit, consequently helping to prevent the development of depression. These considerations should be integrated into the formulation of Thai public health policy. The present research will support the development of tailored and context-specific strategies to address depression and enhance the overall mental health of the Thai population in specific settings.

## Supporting information

S1 Data (XLSX)
